# Smith-Kingsmore syndrome with nystagmus as the initial symptom

**DOI:** 10.1186/s42494-023-00135-2

**Published:** 2023-10-13

**Authors:** Meiling Cai, Yanfei Zhao, He Wang, Shicheng Liu, Huiyi Jiang

**Affiliations:** Department of Pediatric, The First Hospital of Jilin University, Changchun, 130021 China

**Keywords:** *MTOR* gene, Smith-Kingsmore syndrome, Nystagmus, EEG

## Abstract

**Background:**

Smith-Kingsmore syndrome (SKS) is a rare autosomal dominant disorder caused by *de novo* mutations of gene *MTOR* in most cases and germline mosaicism in a few cases. The first case of SKS was reported in 2013. The incidence of SKS remains unknown. The clinical manifestations of SKS are diverse, and common features are macrocephaly, intellectual disability, and seizures. Some patients with SKS have special facial features.

**Case presentation:**

The case was a 5-month-old baby girl, who was admitted to the hospital for nystagmus, delayed development for 2 months, and intermittent convulsions for 2 days. The patient had a head circumference of 42 cm (+ 2SD), and showed facial deformity, low limb muscle tension, large areas of pigmentation, as well as mosaic patchy and strip-like pigment loss in her trunk and limbs. Meanwhile, her development was lagging behind peers. Physical examination did not reveal other abnormalities. She was diagnosed with SKS based on whole-exome sequencing combined with clinical symptoms and signs. She successively received treatment with adrenocorticotropic hormone, methylprednisolone sodium succinate, topiramate, levetiracetam, and zonisamide to reduce the number of convulsions in a short time, but drug resistance appeared thereafter. After combined treatment with multiple antiseizure medications, the patient still had seizures, but the amplitude of limb movement during the seizures was reduced compared to that before treatment.

**Conclusions:**

This case expanded the phenotypic spectrum of SKS for diagnosis. We also review the related literature to promote the awareness, diagnosis, clinical management, and follow-up of SKS patients with *MTOR* mutations.

## Background

Smith-Kingsmore syndrome (SKS) is a rare autosomal dominant disorder with *de novo* mutations of *MTOR* in most cases and germline mosaicism in a few cases. The first case was reported by Smith et al. in 2013 [1]. The incidence of SKS remains unknown. The clinical manifestations of SKS are diverse, and common features are macrocephaly, intellectual disability, and seizures. Some patients with SKS have special facial features.

Here, we report a case of SKS with nystagmus as the initial symptom. The patient was a 5-month-old female who was diagnosed with nystagmus on November 12, 2021. She had developmental delay by 2 months and suffered intermittent convulsions for 2 days. Maternal pregnancy history and birth history showed no abnormalities. Her mother had given births to two babies separately, and the patient was delivered full term. Physical examinations included: head circumference of 42 cm (+ 2SD); facial deformity; low-limb muscle tension; large areas of pigmentation in the trunk and limbs; mosaic, patchy and stripped depigmentation; and development lagging behind peers. The child was diagnosed with SKS according to the results of whole-exome sequencing combined with clinical symptoms and signs. We report this case in the aim to improve clinicians’ awareness of the disease.

## Case presentation

A 5-month-old baby girl was admitted to our hospital for nystagmus, 2-month developmental delay, and intermittent convulsions. She was noted with spontaneous isolated horizontal nystagmus at around 3 months of age, with a frequency of 5-6 times a day and episodic duration of 3-5 s. The incidence and duration of nystagmus increased before admission. During the nystagmic period, her eyeballs were looking downwards accompanied by body twitches. She could not raise her head at age of 3 months, suggesting developmental delay. There was no abnormality during pregnancy or at delivery. Her elder brother had normal development, and there was no noticeable family history of a hereditary disease.

**Fig. 1:**
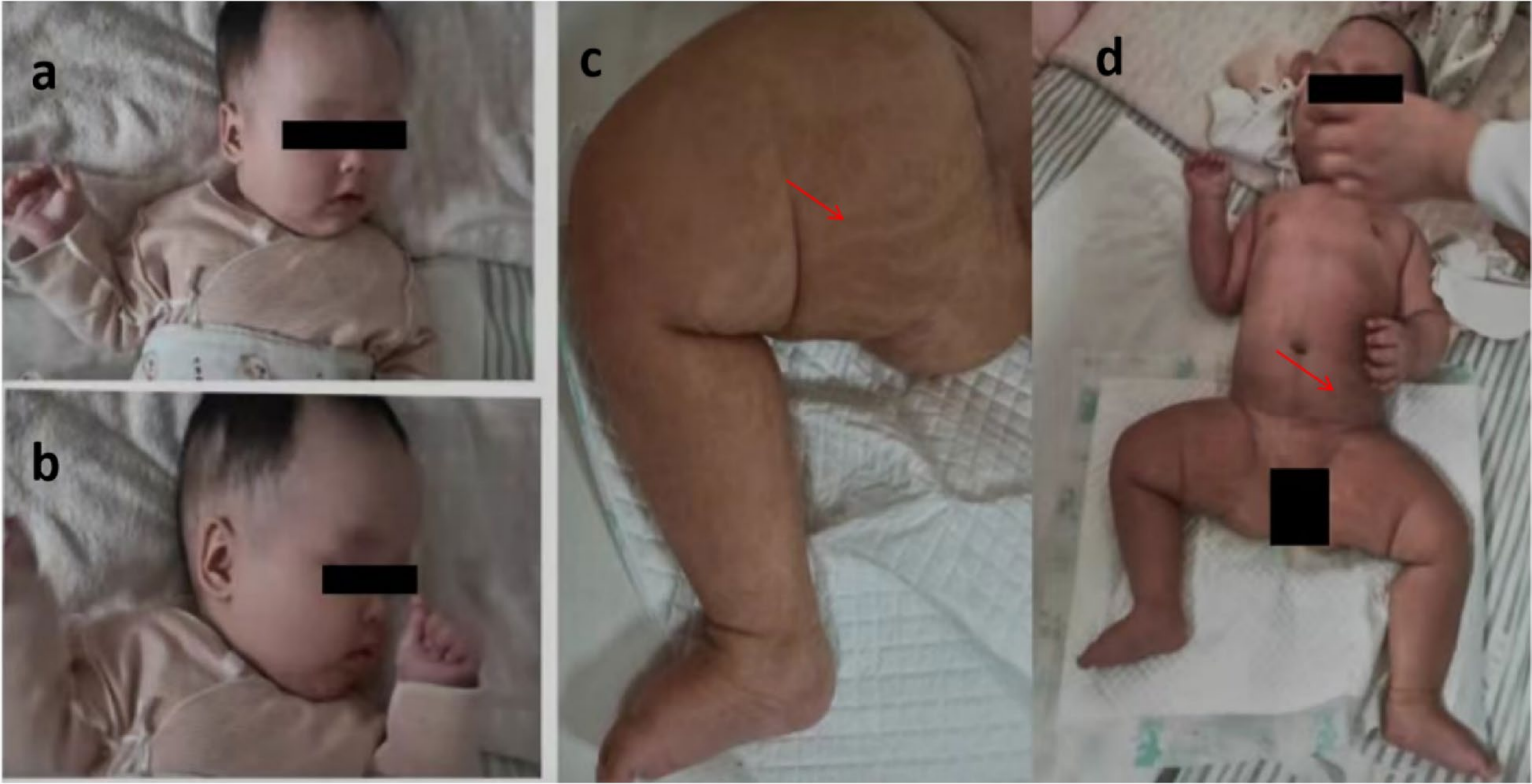
The patient had a prominent forehead, a flat nose bridge, an overly large brain size (**a**, **b**), and café-au-lait spots on the trunk and limbs (**c**, **d**)

At age of 5 months, she was 67 cm in length, weighed 8.5 kg, and had a head circumference of 42 cm (+ 2SD), a protruding forehead, an apparent bregma (approximately 4 * 4 cm^2^ in size), a low and flat nose bridge, sloping eye fissure, and a small mandible. Abnormal, movable limbs, and low muscle tone, with negative pathological signs, were also observed. Large areas of pigmentation and mosaic, patchy, strip-like depigmentation were found on the trunk and limbs with café-au-lait spots (Fig. 1).

**Fig. 2:**
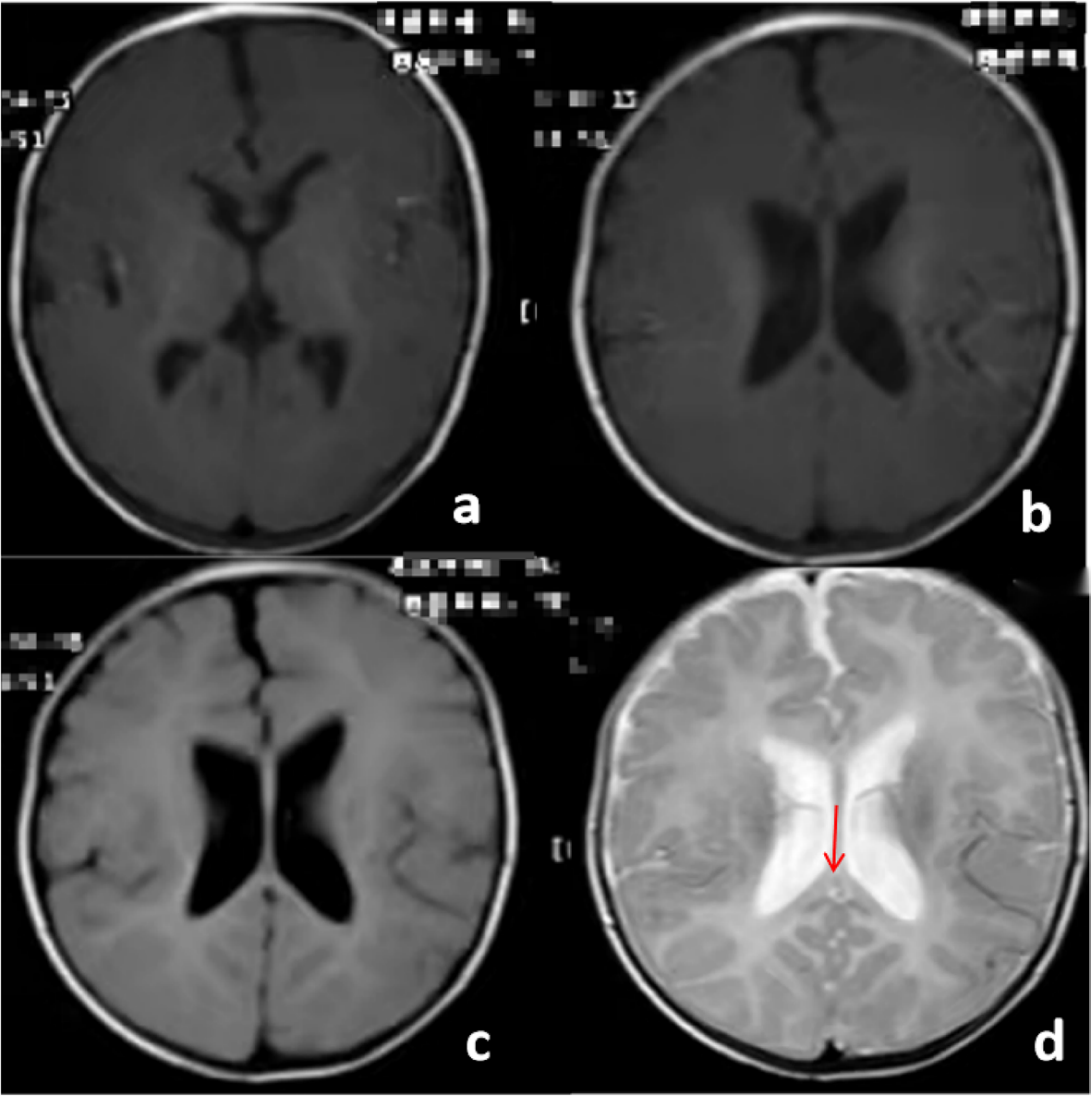
Brain MRI revealed dysplasia of the corpus callosum and bilateral ventricle dilatation (**a**-**d**)

The Denver developmental screening test revealed a Mental Index of 58 and a Developmental Quotient of 47, indicating a developmental delay (equivalent to age 1 month). Brain magnetic resonance imaging (MRI) showed dysplasia of the corpus callosum and bilateral ventricle dilatation (Fig. 2).

### Karyotype analysis

Karyotype analysis showed that the patient had two X chromosomes (46, XX). No abnormal chromosome structure was found.

### Whole-exome sequencing

**Fig. 3:**
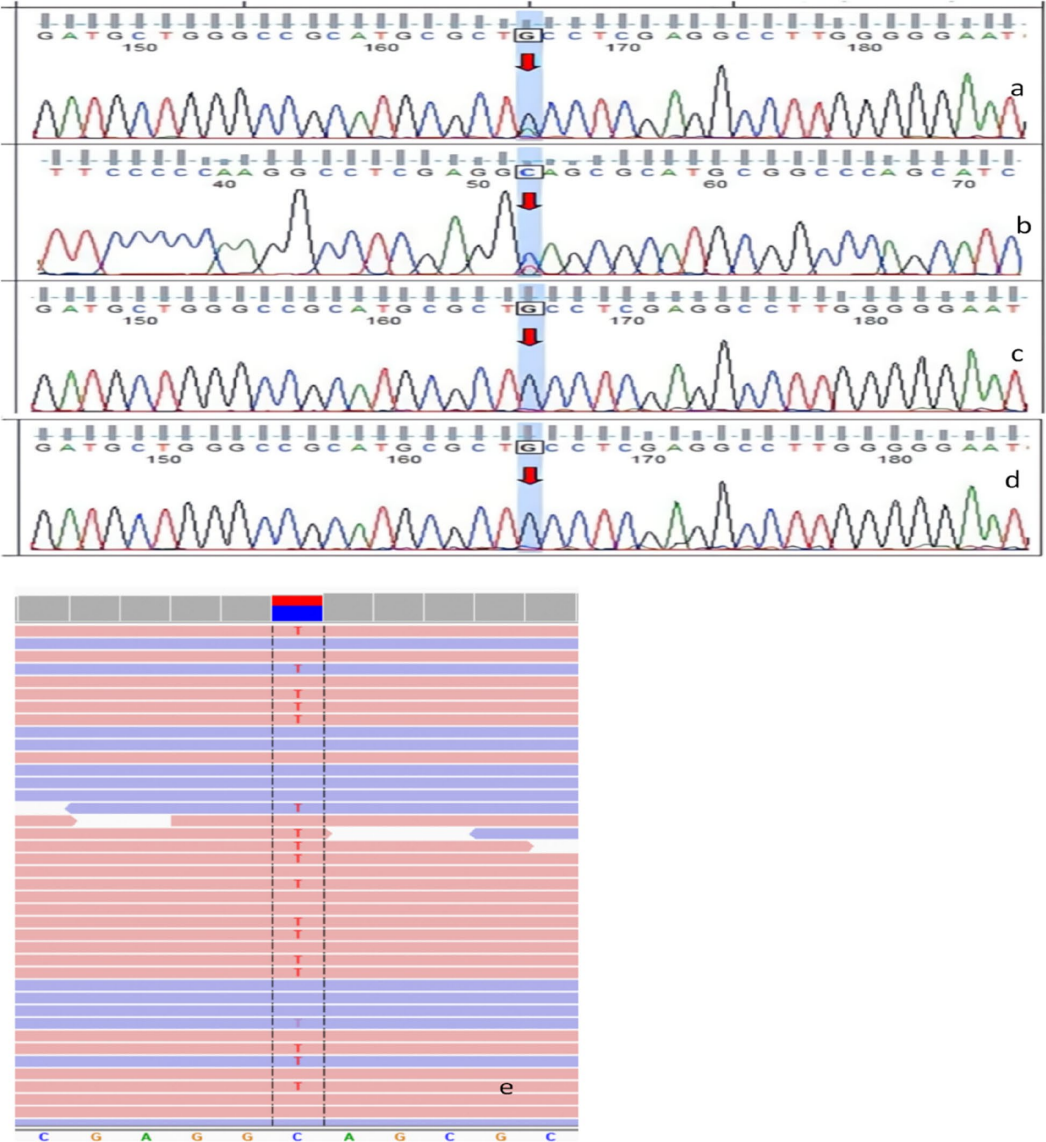
Whole-exome sequencing of *MTOR*. **a** Sanger sequencing of the proband-forward sequencing; (**b**) Sanger sequencing of the proband-reverse sequencing; (**c**) sequencing of mother-wild-type; (**d**) sequencing of father-wild-type; (**e**) next-generation sequencing revealed chr1:11217230 Exon30 NM 004958 4c.4448G>A (pCys1483Tyr) mutation in *MTOR*. The bottom sequence is the genome hg19 reference sequence; the variant site is displayed between the black dotted lines

A missense mutation was detected at chr1:11217230 Exon30 NM_004958.4 (*MTOR*):c.4448G>A (p. Cys1483Tyr) (Fig. 3). According to the guideline of the American College of Medical Genetics and Genomics (ACMG), the variant locus was classified as a pathogenic variant (PM2_P, PP2, PM5, PM1_P, PS4, PS2).

Video electroencephalography (VEEG) performed continuously for 6 h revealed abnormal brain activities during both awake and sleeping periods. Eleven solitary attacks and two clusters of spastic-seizure episodes were identified during VEEG recording.

**Fig. 4:**
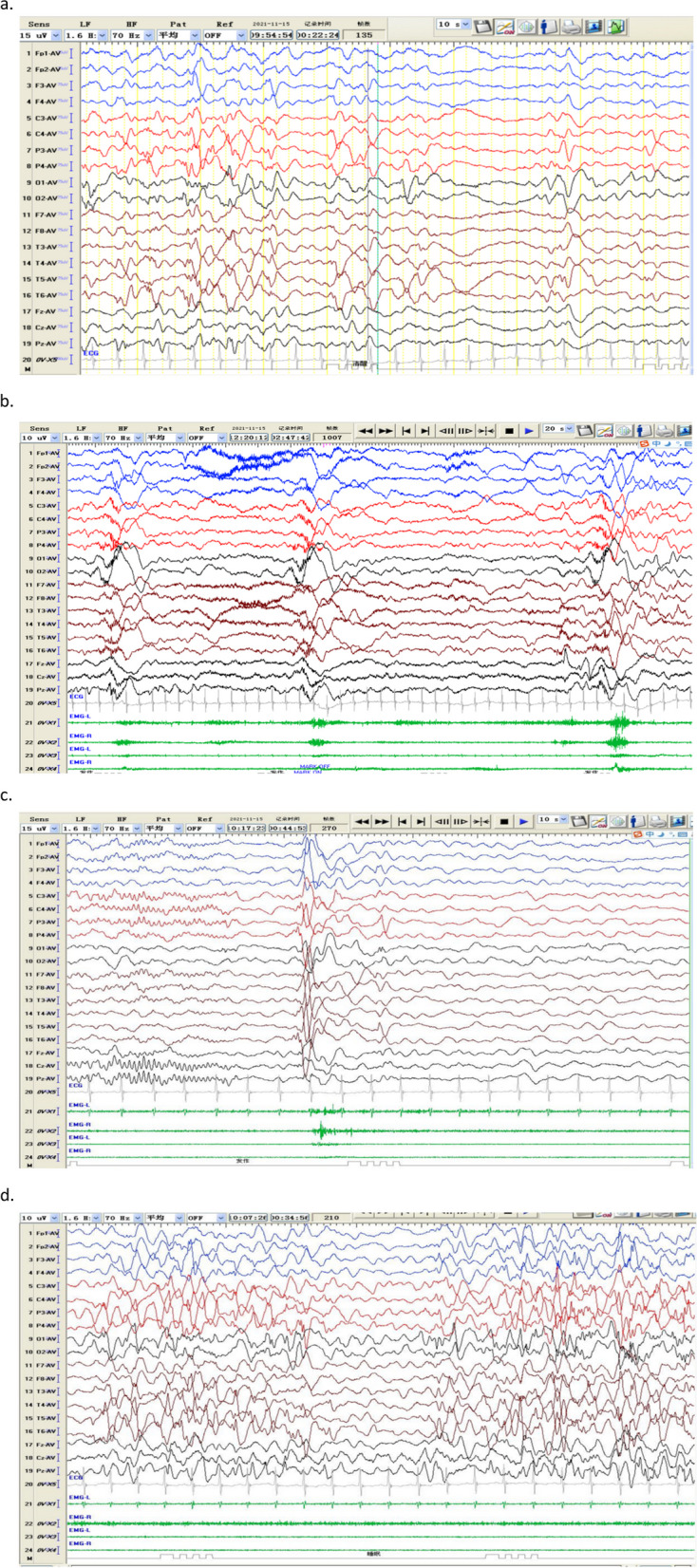
Abnormal EEG samples at (**a**) awake time with no seizure; (**b**) awake time with a seizure; (**c**) sleeping time with a seizure event; and (**d**) sleeping time with no seizure

Normal EEG was recorded only for one-third of the awake time; during the rest of the time, hypsarrhythmia, multiple waves of medium-to-high amplitudes, multiphases and slow waves were detected widely at many lead sites (Fig. 4a). Solitary spikes and clustered spastic seizure-like waves were also seen. During a solitary attack, the spastic episodes were identified as head nodding, eyes looking downward, and limb flexion. EEG recording showed low-amplitude fast waves at the back of the head, followed by medium-to-high-amplitude slow-wave bursts for 1-2 s. During seizure clusters, multiple spastic events appeared as head nodding (10-20 times), eyes looking downward, and limb flexion. EEG recording during this period showed fast waves at the back of the head, followed by widely distributed, high-amplitude, polyphasic waves (Fig. 4b). Generalized voltage drops (4-6 s) were also seen after seizure events. Widely distributed, high-amplitude, sharp, slow-wave bursts were also observed.

Six single spastic seizure events were recorded within 1 h during sleep, accompanied by head nodding, eyes looking downward, and limb flexion. However, there were more distributed high-amplitude peak waves, slow-wave bursts, and electromyography (EMG) bursts during sleep, compared to those recorded during the awake time (Fig. 4c).

During sleep, when there was no spastic seizure event, abnormal EEG findings were also apparent, including medium-to-high-amplitude waves, spike waves, focal spikes, and poly spike waves at various lead sites (Fig. 4d).

EEG performed at the age of 6 months also showed hypsarrhythmia at sleep onset, including sharp waves, spike waves, sharp and slow waves, and slow waves in the bilateral occipital regions. The spastic seizures were accompanied by nonmotor focal seizures, with onset in the right Rolandic area.

### Treatment

During hospitalization, the patient was successively treated with adrenocorticotropic hormone, methylprednisolone sodium succinate, topiramate, vitamin B6, human immunoglobulin, and levetiracetam. The number and amplitude of convulsions were significantly reduced, but drug resistance developed. After discharge, oral therapy of methylprednisolone sodium succinate, zonisamide, and levetiracetam was continued. The patient was followed up by telephone interview and regular outpatient visits. The overall impression of this treatment was far from idealy.

## Discussion

SKS is an emerging disease, with the first case being reported in 2013 [1]. Moosa et al. [2] later identified a family of children with *MTOR* mutation c.5395G>A p.(Glu1799Lys), in which the old siblings showed occurrence of multiple intestinal polyps. Gordo et al. [3] reported four new cases of SKS in 2017 and summarized the clinical characteristics of the patients with brain somatic mutations in *MTOR*.

In 2019, Elena et al. [4] reported a patient who had *MTOR* mutation (c.7235A>T: p.(Asp2412Val)) and exhibited SKS and antiphospholipid syndrome, which further expanded the phenotypic spectrum of *MTOR*-related diseases. Lee et al. [5] reported a case of SKS with *MTOR* germline mutation c.5395G>A p.(Glu1799Lys) in Korea.

In 2020, Anasofia et al. [6] reported a 5-year-old patient with a heterozygous *MTOR* mutation c.5663T>G (p.Phe1888Cys). The patient had not experienced seizures but presented bilateral cataracts. Carli et al. [7] reported a 7-year-old boy with SKS, who possessed a somatic *MTOR* pathogenic variant resulting in lateralized overgrowth.

The pathogenic variant described in our case is consistent with the case reported by Carli et al. in a previous study [7]. The 7-year-old case reported in that study had facial features, hypomelanosis of Ito on the left side of the body, left hemicerebral malformation in transverse and coronal positions on brain MRI, and lateralized overgrowth on the left side of the body with a longer left leg. This patient possessed the same *MTOR* mutation as our case (c.4448G >A, p.Cys1483Tyr), which is ultimately identified as a chimeric system mutation. The *MTOR* pathogenic variant was proposed to be a cause of asymmetric body overgrowth. Both cases had facial deformities, hypomelanosis of Ito, and abnormal brain MRI. However, our case did not show asymmetric body growth, thus follow-ups are needed to monitor whether this symptom would develop in our case. In addition, our case was younger at the onset of the disease and exhibited nystagmus that had never been described before. The *MTOR* pathogenic imposex may be a cause of nystagmus.

In 2021, Szczałuba et al. [8] performed histopathological analysis of the brain tissue after surgery in a patient with SKS caused by a low-level *MTOR* chimeric mutation. The patient showed recurrent hypoglycemia, which may be related to the interrupted PI3K-AKT-mTOR signaling pathway.

Poole et al. [9] investigated 16 previously unreported and reported cases and reported that neurocognitive deterioration, sleep disturbance, and hypoglycemia may be the main clinical manifestations of SKS. An evidence-based management guidance for SKS was proposed, listing the clinical features of SKS such as intellectual disability, head circumference > +3 SD, behavioral problems, speech delay, brain MRI abnormalities, neonatal hypotonia, neurocognitive/behavioral deterioration, vascular abnormalities, sleep disturbance, postpartum hypotonia, afebrile convulsions, strabismus, gastrointestinal problems, joint hypermobility, neonatal feeding difficulties, hypoglycemia, and scoliosis.

The nystagmus exhibited by our case may add to the indications for an early diagnosis of SKS. Therefore, in the presence of diverse clinical manifestations accompanied by nystagmus, a genetic testing for SKS is recommended to improve the diagnosis of SKS.

Møller et al. [10] reported two patients with clonic eye movements. In 2018, Gordo et al. [3] reviewed the clinical characteristics of all previously reported clinical cases and their four cases, including cases with visual problems. Among these patients, ten had strabismus and three had visual impairment. However, none of the patients with pathogenic *MTOR* variants were reported to have nystagmus. In our case, the patient presented nystagmus consistent with congenital nystagmus (CN). CN is an involuntary, rhythmic, binocular eye shock that usually occurs within 6 months after birth, often accompanied by amblyopia, strabismus, and abnormal head positioning. In our case, nystagmus was the first symptom that prompted her parents to seek medical treatment. Thus, this case may also notify primary care providers to expand their examination and conduct genetic tests to verify SKS. Nystagmus and other abnormal eye movement may be sensitive indications for SKS diagnosis.

Some manifestations of SKS may indicate structural and functional abnormalities of the brain, including neonatal hypotonia, neurocognitive/behavioral deterioration, sleep disturbance, postpartum hypotonia, and afebrile convulsions [9]. Brain MRI may reveal structural alterations. The abnormalities may extend to ocular motor control circuits throughout the brainstem [11], causing nystagmus and other eye movement disorders.

The PI3K-AKT-mTOR signaling pathway integrates intracellular and extracellular signals and is the central regulator of cell metabolism, growth, proliferation, and survival [12]. *MTOR* mutations affect the expression of the corresponding protein in the *mTOR* signaling pathway, resulting in different clinical manifestations. Mutations of the *mTOR* pathway genes or abnormal activities of the mTOR pathway can cause epileptic activity in the brain [13]. Some inflammatory mediators such as IL-1β and reactive oxygen species can participate in epileptogenesis through the mTOR pathway. The mTOR pathway is critical for biological processes of the central nervous system, including cortical development, axonal and dendritic morphology, immune responses, neurotransmitter expression, ion channel expression, synaptic plasticity, cognition, and behavior. Disturbance of the mTOR pathway and subsequent abnormalities of the above biological processes can contribute to the development of epilepsy [14].

In the presence of *mTOR* mutation, the balance between protein synthesis and mitochondrial activity is also disrupted, resulting in deficiency of oxidative phosphorylation and oxidative stress. The mitochondria-specific phospholipid cardiolipin is present on the surface of apoptotic cells and may trigger antiphospholipid syndrome [4].

The synaptic plasticity in the cerebral cortex may be highly affected by the mTOR pathway. Circadian rhythms may also be affected. Activation of the mTOR pathway increases the level of canonical clock proteins, represented by the translation factor BMAL1 [15]. Therefore, *MTOR* mutations may cause neurocognitive deterioration and sleep disturbance.

Previous reports have provided a highly diverse phenotypic profile of SKS. Hypomelanosis of Ito can be present early as linear or plaque hypopigmentation, together with central nervous system abnormalities. Carmignac et al. [16] described hypomelanosis of Ito associated with *MTOR* mutations. However, here, our case was too young to undergo most of the clinical, biochemical and genetic evaluation. EEG and MRI were the main assessment tools for brain abnormalities. For this patient, our therapeutic strategy was focused on management of epilepsy.

The nystagmus may also be related to retinal problems, such as the lack of retinal pigment epithelium and photoreceptor damage [17, 18]. A recent study showed that MTOR proteins might also affect retinal pigment epithelial cells [19].

*MTOR* c.5395G>A p. (Glu1799Lys) is the most common missense mutation for SKS. The most common clinical manifestations of SKS are developmental retardation or mental retardation with varying (more commonly moderate to severe) degrees of developmental retardation, and more prominently, language development disorders. Some patients will display developmental regression. The most specific manifestation of SKS is the large brain size, with the head circumference increasing rapidly in the early stage of life, and then the growth rate gradually becomes normal, resulting in a significantly greater head circumference than the peers. Facial developmental malformations are also common, including wide eye distance and a flat nose bridge. In addition, SKS also has extracerebral manifestations such as hypoglycemia, and lateral limb overgrowth. The characteristic developmental delay, greater head circumference and seizures of our case are consistent with previous reports, and she did not show hypoglycemia, lateral limb overgrowth, antiphospholipid syndrome or other manifestations. Our case was the youngest child to seek medical attention, with an unreported clinical presentation, nystagmus, which expands the clinical symptoms of SKS. The lack of literature report of nystagmus in SKS may be related to the young age and small number of patients. It remains to be observed in clinic in the future.

## Conclusions

This report mainly aimed to present a case of SKS with nystagmus. Although this patient had extensive abnormalities in EEG and body movements, nystagmus was the first warning sign that prompted her parents to seek medical treatments. Our report expands the phenotype spectrum of SKS. Attention should be paid to nystagmus at diagnosis of this disease. While genetic testing was necessary for confirming the diagnosis, eye movement abnormalities could be a potential indicator for SKS.

## Data Availability

The datasets analyzed in this study are available from the corresponding authors on reasonable request.

## Abbreviations

CN: Congenital nystagmus
EEG: Electroencephalography
SD: Standard Deviation
SKS: Smith–Kingsmore syndrome
VEEG: Video electroencephalography
